# Evaluation of the diagnostic performance of self-navigated whole-heart contrast-enhanced coronary MRA at 3T

**DOI:** 10.1186/1532-429X-18-S1-P361

**Published:** 2016-01-27

**Authors:** Yi He, Jianing Pang, Zhanming Fan, Jing An, Tianjing Zhang, Debiao Li

**Affiliations:** 1 grid.411606.40000000417615917Beijing Anzhen Hospital, Beijing, China; 2 Collaborations NE Asia, Siemens HealthcareMR, Beijing, China; 3 grid.50956.3f0000000121529905Cedars Sinai Medical Center, Los Angeles, CA USA

## Background

Recently, a self-navigated whole-heart coronary MRA technique has been developed to address these limitations of conventional coronary MRA techniques. Our study was to evaluate the diagnostic performance of self-navigated whole-heart coronary magnetic resonance angiography (CMRA) at 3T, using conventional invasive coronary angiography (ICA) as the reference.

## Methods

60 consecutive patients underwent CMRA, 39 of which later underwent ICA. CMRA was performed on a 3T clinical scanner during free-breathing using an ECG-gated, fat-saturated, inversion-recovery prepared spoiled gradient-echo sequence with 3D radial k-space trajectory, self-navigated motion correction, and offline non-Cartesian sensitivity encoding reconstruction. The CMRA images were evaluated by two experienced readers to detect significant luminal narrowing (>50% diameter reduction).

## Results

All patients completed CMRA successfully, with one excluded from analysis (1.6%, due to poor image quality). From the 59 included patients, a total of 506 coronary segments were evaluated. In addition, 39 of the 59 patients underwent ICA, where 315 of 367 (85.8%) segments with a reference luminal diameter ≥1.5 mm were assessable on CMRA. The sensitivity, specificity, positive predictive value, negative predictive value, and accuracy on a per-patient basis were 81.8%, 81.3%, 85.7%, 76.5%, and 81.6%, respectively.

## Conclusions

Contrast-enhanced self-navigated CMRA on 3T is a promising technique for the noninvasive detection of significant coronary stenosis, and future technical improvement efforts are warranted to make it clinically viable.

**Figure 1:**
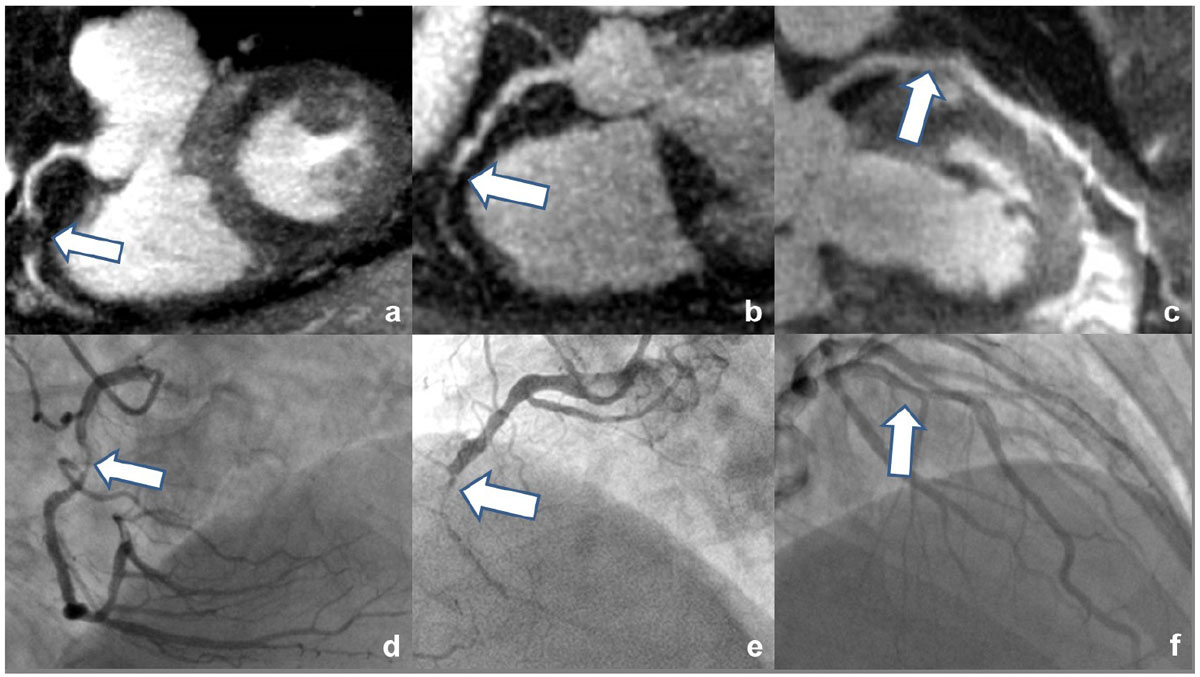
**coronary MRA multi-planar reformats with significant stenosis**.

**Figure 2:**
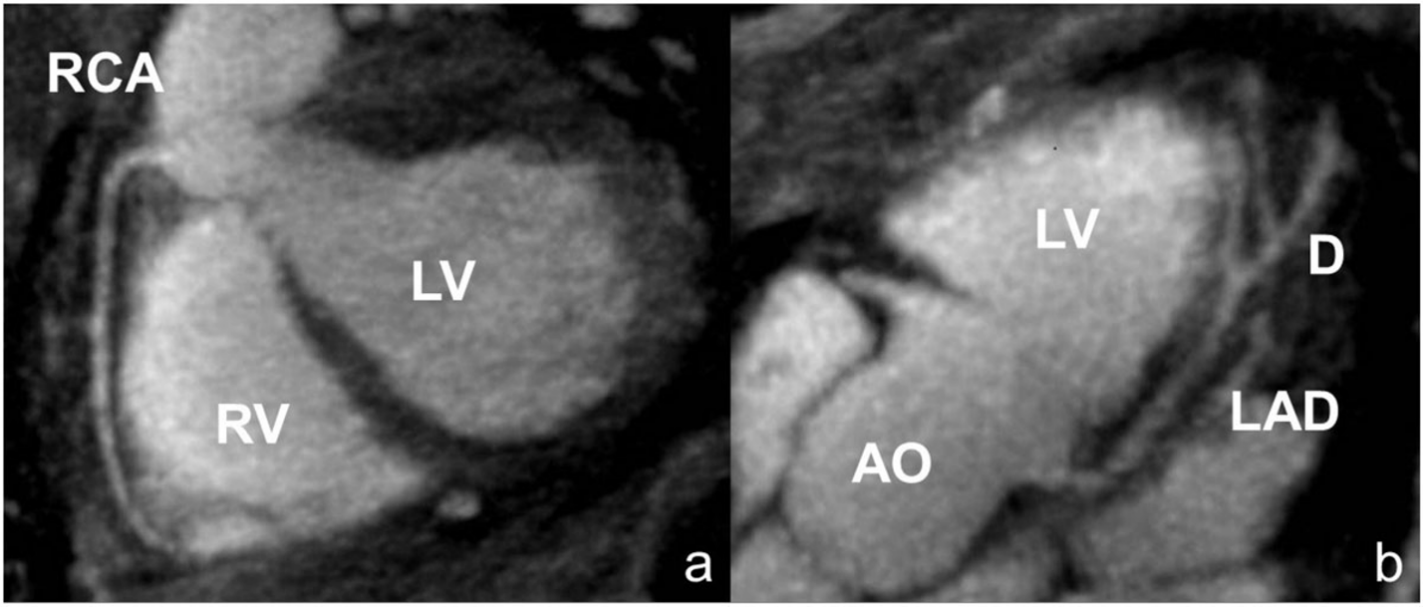
**Representative multi-planar reformats of the coronary arteries: (a) normal RCA in patient with suspected CAD; proximal and middle segments were clearly seen; (b) left main, proximal and middle segment of LAD**.

